# Complete replantation of a small tissue segment at the auricular apex: A case report

**DOI:** 10.1016/j.jpra.2025.09.032

**Published:** 2025-10-06

**Authors:** Haiyuan Han, Longxin An, Jie Zhao, Xuecheng Sun, Futian Zhang, Naibo Feng

**Affiliations:** a Department of Trauma Orthopedics, Weifang People's Hospital, Shandong Second Medical University, Weifang 261000, China; b School of Clinical Medicine, Shandong Second Medical University, Weifang 261042, China

**Keywords:** Ear replantation, Supermicrosurgical anastomosis, Venous congestion, Auricular apex avulsion

## Abstract

This study reports a rare case of complete replantation of a small avulsed tissue segment at the auricular apex. The patient, a 30-year-old male, sustained a complete amputation of the left ear apex due to a traffic accident. The avulsed tissue had been ischemic for approximately 4 h prior to surgery. Using supermicrosurgical techniques under 30 × magnification, three vessels—one artery and two presumed veins, each with a diameter of approximately 0.2–0.4 mm—were successfully anastomosed. Postoperatively, the patient developed venous congestion, which was effectively managed through regular acupuncture bleeding, leading to restored venous outflow. The tissue survived completely, and during a 3- to 6-month follow-up, the patient showed no significant hearing loss, with good auricular contour and near-normal sensory function. This case suggests that, under appropriate microsurgical conditions, even small tissue segments at the ear apex may be considered for vascular replantation. Accurate intraoperative vessel identification and active management of postoperative venous crisis are critical to success.

## Introduction

The external ear, being a prominent structure on the craniofacial contour, is highly susceptible to trauma from bites, shearing, and other injuries due to its exposed location. Loss of the auricle not only compromises cosmetic appearance and hearing function but also imposes significant psychological stress on the patient. Since the first successful vascular anastomosis in ear replantation was reported in 1980,^1^ numerous cases of total auricular replantation have been published.^2^, ^3^, ^4^ However, successful replantation of partial auricular segments, especially the auricular apex, remains rarely reported. In this case, a small avulsed tissue block at the auricular apex survived completely after vascular anastomosis, with no similar reports found in the literature.

## Case presentation

**Figure 1:**
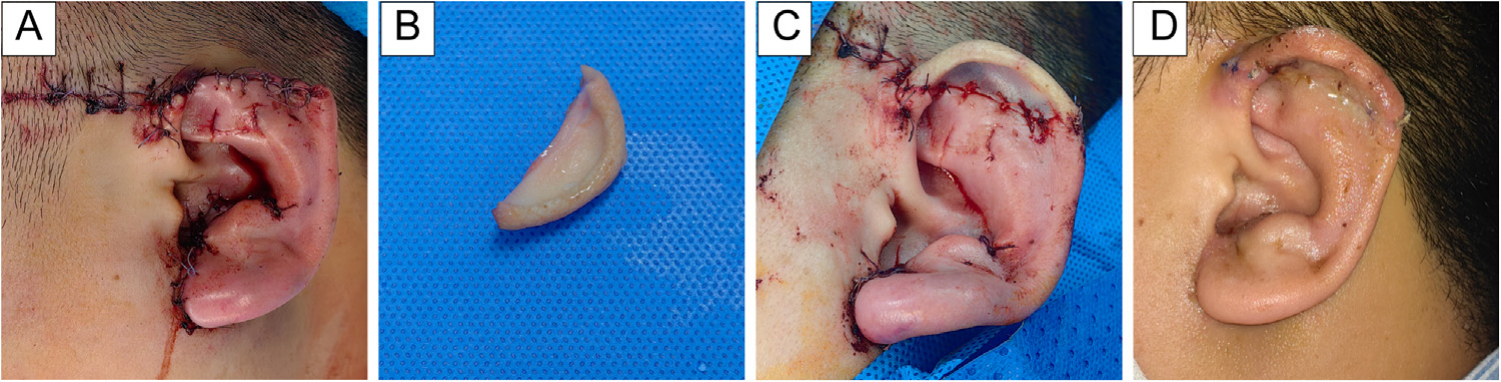
(A) Patient after initial debridement and suturing at the local hospital. (B) Avulsed auricular segment (2.5 cm × 1.2 cm). (C) Immediate postoperative appearance. (D) Appearance at postoperative day 30.

A 30-year-old male presented with complete avulsion of the left auricular apex due to a traffic accident. Initially, he received wound closure at a nearby hospital as the avulsed tissue was missing. Approximately 4 h later, the tissue segment (2.5 cm × 1.2 cm) was recovered at the scene and the patient was transferred to our facility. Physical examination showed upper auricular defect with a sutured stump and multiple facial lacerations (Figure 1A and B, Supplementary Figure 1A).

## Surgical procedure

At 6 h post-injury, the patient underwent emergency surgery under general anesthesia. The avulsed segment was soaked in heparinized saline (12,500 U/500 mL) for 10 min and debrided under a supermicroscope. Under 30 × magnification, careful exploration identified two veins (diameter ∼0.3–0.4 mm) on the dorsal one-third of the auricle and one artery (∼0.2 mm) on the medial cheek-facing side. These vessels were marked using 10–0 suture.

After removing initial sutures, the wound was irrigated. Auricular cartilage was aligned and sutured with 5–0 absorbable sutures, and the auricle temporarily fixed to expose the posterior surface. Under microscopy, proximal vessel stumps were identified and clamped. Two dorsal veins were anastomosed with 11–0 sutures (six stitches each) (Supplementary Figure 1B), followed by medial artery anastomosis with 12–0 sutures (four stitches) (Supplementary Figure 1C). Perfusion was confirmed with mild capillary refill and low tension (Figure 1C). Skin closure completed the 4-h procedure with ∼20 mL blood loss. Heparin sodium (12,500 U in 500 mL 0.9 % saline) was infused intravenously at 7 drops/min once daily for 7 days. To improve microcirculation and prevent vasospasm, papaverine hydrochloride (30 mg in 100 mL saline) was administered by IV drip at 30 drops/min every 6 h for 7 days. Cefuroxime (1.5 g in 250 mL saline) was given at 30 drops/min every 8 h for 3 days for infection prophylaxis, during which no signs of infection were observed. Local warming was maintained with a medical infrared lamp (800–1000 μm, ∼40 cm from the auricle) applied continuously for 24 h daily over 7 days, keeping the replanted ear at 32–34 °C.

## Postoperative course

At 6 h postoperatively, the auricle appeared dark and tense (Supplementary Figure 1D), indicating venous congestion. Needle-prick bleeding released dark blood initially, followed by bright red blood after 10 s, with gradual improvement in skin color. Needle-prick bleeding was performed with a 21 G needle every 2 h at 2–3 sites, with continuous irrigation using heparinized saline (12,500 U/500 mL). After fresh blood appeared, bleeding was maintained for an additional 20 s. (Supplementary Figure 1E, Supplementary video 1).

After 48 h, venous outflow stabilized but congestion persisted (Supplementary Figure 1F). The same protocol was continued. From postoperative day 5, needle-prick bleeding was reduced to every 4 h and limited to 10 s per session, and discontinued by day 7. Although bleeding volume could not be quantified, the protocol was to continue 20 s after fresh blood appeared. After day 5, fresh blood emerged immediately on puncture (Supplementary Figure 1 G), allowing shorter sessions. By day 7, edema had further subsided, and wound healing was satisfactory (Supplementary Figure 1H). Anticoagulants and vasodilators were discontinued, and the patient was allowed to ambulate indoors.

By postoperative day 30, the replanted auricle was viable with minimal difference in color and temperature compared to the contralateral ear. Sutures were removed, and the ear had fully survived (Figure 1D). At 3-month follow-up, the patient exhibited preserved ear shape and hearing with no atrophy. Sensory function was mildly reduced. At 6 months, the replanted auricular apex showed stable cartilage, maintained contour and volume, with normal texture, elasticity, and near-normal pain and temperature sensation.

## Discussion

Microsurgical reconstruction of auricular blood supply is well established.^1^, ^2^, ^3^, ^4^ However, replantation of the auricular apex remains technically demanding due to the tiny vessel diameters (∼0.3 mm) and anatomical variability. Identifying suitable veins for anastomosis is particularly challenging, often complicating both the surgery and postoperative care.^5^ Successful replantation depends critically on early identification of viable vessels. In this case, meticulous debridement and microscopic examination prior to fixation allowed precise localization and marking of distal vessels. This facilitated efficient alignment with proximal counterparts after cartilage fixation, improving accuracy and reducing operative time. The auricle is richly vascularized by branches of the posterior auricular and superficial temporal arteries, forming anterior and posterior arcades with extensive interconnections. Understanding this vascular anatomy is essential when planning vessel selection and anastomosis during partial auricular replantation.^6^^,^^7^

Although prior studies suggest limited availability of suitable arteries in the upper auricle,^5^ we successfully identified and anastomosed a ∼0.2 mm artery and two additional ∼0.4 mm vessels. According to Weerda’s classification, this represents a third-degree auricular defect.^8^ Given the small tissue size and unpredictable vascular anatomy, it is advisable to anastomose all viable vessels rather than insist on strict artery–vein pairing. Momeni et al. found no significant difference in outcomes between arterial-only and artery–vein reconstructions across 60 ear replantation cases.^9^

Perioperative management is equally important. Patients undergoing auricular replantation are particularly prone to venous congestion, which, if unmanaged, can compromise flap survival. Pertea et al.^10^ reported three cases of total ear amputation treated with arterial-only anastomosis and early application of medicinal leech therapy to relieve venous congestion, achieving favorable outcomes. While leech therapy has shown benefits, it carries risks of severe infection.^5^ Instead, needle-prick bleeding has proven to be a safer alternative with comparable efficacy. In our case, the patient developed signs of venous congestion at approximately 6 h postoperatively. Controlled needle-prick bleeding was initiated and continued regularly for 5 days, leading to gradual resolution of congestion and successful venous return. Precise cartilage alignment with absorbable sutures, protection from external compression, and adequate revascularization were key to successful healing in this case. Supermicrosurgical arterial anastomosis combined with controlled needle-prick bleeding maintained an aerobic environment, preventing chondrocyte apoptosis and excessive resorption. Consistent with Fan et al.,^11^ prolonged ischemia markedly increases cartilage loss, underscoring the importance of timely revascularization. Together, these measures ensured stable cartilage support, favorable contour, and near-normal function after auricular apex replantation.

## Conclusion

This case confirms the feasibility of complete replantation of small auricular apex segments using supermicrosurgical techniques. Vessels as small as 0.2–0.4 mm can be successfully anastomosed with reliable perfusion. Key factors for success include meticulous vessel identification, precise technique, and proactive postoperative management—particularly early intervention for venous congestion. This case broadens the clinical indications for vascularized replantation in partial ear amputations, especially for small, distal segments.

## Funding

This work was supported by the 10.13039/501100001809National Natural Science Foundation of China (No. 82302031), the 10.13039/501100007129Natural Science Foundation of Shandong Province (No. ZR2024QH033), Weifang Science and Technology Development Project (No. 2023RKX158).

## Ethical approval

This study was approved by the Ethics Committee of the First Affiliated Hospital of Shandong Second Medical University (Approval No KYLL20250721-5). Informed consent was obtained from the patient in this study.

## Declaration of competing interest

None.

## Appendix Supplementary materials

Image, application 1

Image, application 2
