# Supplementary material for: Complete replantation of a small tissue segment at the auricular apex: A case report
Source: JPRAS Open. 2025 Oct 6;46:446–9. doi: 10.1016/j.jpra.2025.09.032 (PMC12613026; doi:10.1016/j.jpra.2025.09.032)
Supplement: Supplementary file 1 [file mmc1.docx]

**
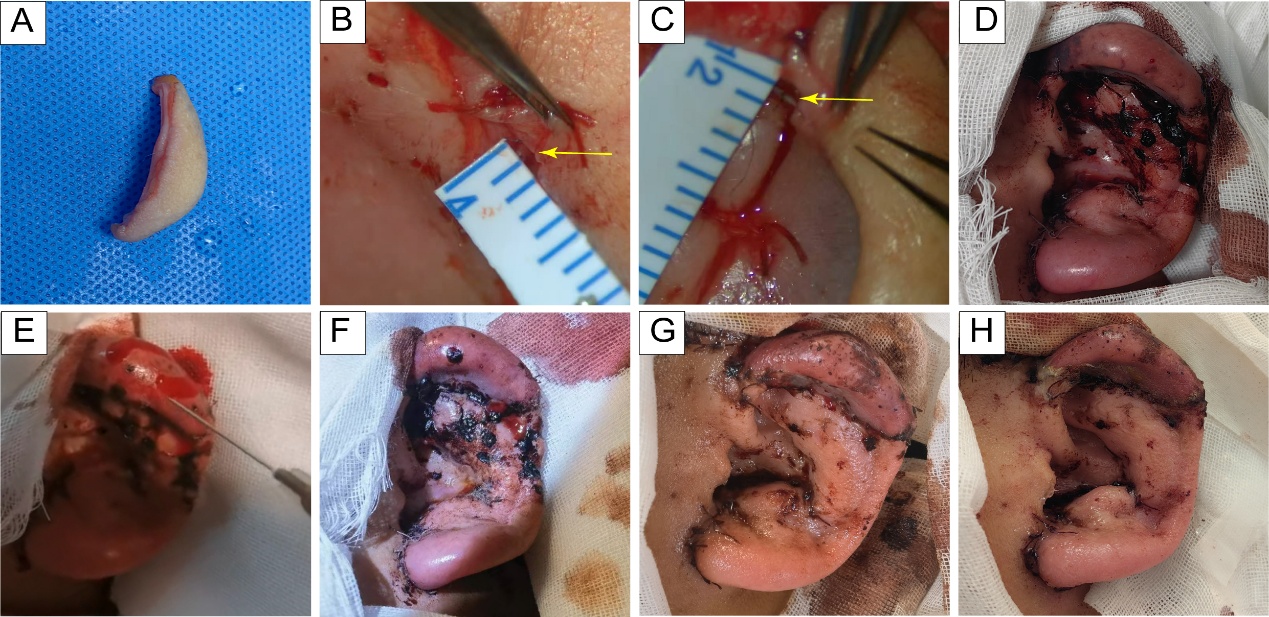
**

**Supplementary Fig. 1.** (A) Avulsed auricular segment (2.5 cm × 1.2 cm). (B) Intraoperative anastomosis of an artery (~0.2 mm in diameter), likely a superior branch of the superficial temporal artery. (C) Intraoperative anastomosis of a vessel (~0.4 mm in diameter; artery or vein undetermined). (D) Appearance at 6 hours postoperatively showing dark discoloration and increased tension, suggestive of venous congestion. (E) Needle-prick bleeding therapy administered. (F) Appearance at 48 hours postoperatively. (G) Appearance at postoperative day 5. (H) Appearance at postoperative day 7.
